# Risk Factors of Young-Onset Colorectal Cancer: Analysis of a Large Population-Based Registry

**DOI:** 10.1155/2022/3582443

**Published:** 2022-02-16

**Authors:** Daneshvar Danial, El Douaihy Youssef, Bayat Mokhtari Maryam, Abureesh Mohammad, Bayat Mokhtari Moein, Deeb Liliane

**Affiliations:** ^1^Staten Island University Hospital, Gastroenterology Department, Staten Island, NY, USA; ^2^Larkin Community Hospital, Family Medicine Department, South Miami, FL, USA; ^3^Staten Island University Hospital, Internal Medicine Department, Staten Island, NY, USA; ^4^St. George's University, Medicine St. Georges, West Indies St. George, True Blue, Saint George's, Grenada

## Abstract

**Background:**

As the third most common type of cancer in the United States, colorectal cancer (CRC) was previously thought to be rare in young populations. Despite a decrease in the overall incidence of CRC, the rate of new cases under 50 years old has been continuously increasing.

**Aim:**

The purpose of our study was to analyze risk factors of young-onset CRC.

**Methods:**

Commercially available software platform, Explorys, was used to extract data from a collective healthcare database electronically.

**Results:**

In this database, 13,800 young adults (age 20–50) were diagnosed with primary colorectal malignancy. Compared to subjects with a previous family history of CRC who had an odds ratio of 17.78, those diagnosed with primary malignant neoplasm of breast and inflammatory bowel disease (ulcerative colitis and Crohn's) had odds ratios of 16.94, 4.4, and 3.7 for young-onset CRC, respectively. Patients with a history of alcohol abuse, smoking, obesity, diabetes mellitus, and hyperlipidemia had higher chances of developing young-onset CRC. In addition, the odds of CRC were lower in Hispanic ethnicity in comparison to Caucasians (OR: 0.54), with no statically significant differences between Caucasian, African American, and Asian populations.

**Conclusion:**

Currently, this is an expansive study investigating the risk factors for early-onset CRC. The analysis showed factors such as family and individual history of IBD to have high association with early onset. Notably, an individual history of breast malignancy was strongly associated with early-onset CRC.

## 1. Introduction

Colorectal cancer (CRC) is ranked as the third most common malignancy and cause of mortality in men and women. CRC was responsible for 145,600 of newly diagnosed cases of malignancy, and 51,020 deaths in 2019 [1]. Over the past decade, CRC incidence and related mortality in the United States have been decreasing in individuals over the age of 50, which is namely the result of higher uptake of CRC screening. In contrast, incidence and mortality rates have been showing a significant upward trend in those younger than 50 years old [2, 3].

Following this marked increase in CRC, particularly in young individuals, American Cancer Society (ACS) proposed to decrease the age threshold of CRC screening to 45 years for the average-risk population [4], and also American College of Gastroenterology suggested that starting CRC screening at the age of 45 will reduce the mortality from CRC [5].

Considering the multifactorial nature of this neoplasia, the occurrence of CRC is noted to be irregular in the general population and showed considerable variation with several risk factors including genetic susceptibility, obesity, vitamin D deficiency, diabetes, smoking, and other dietary factors [6]. In young adults, scarce and conflicting data is available with regard to underlying predisposing factors for developing early-onset colorectal cancer. Some studies reported nonmodifiable risk factors, such as sex, race, family history of CRC, and inflammatory bowel disease (IBD), to be associated with developing early adult-onset CRC. However, no significant correlation was established with other risk factors, such as diabetes mellitus, alcohol intake, smoking, folate, fiber intake, or vitamin deficiency in young adults [7, 8]. Contrarily, a European case-control study of 329 patients, who were younger than 45 years old, demonstrated that family predisposition, usage of alcohol, and a diet centered on high levels of processed meat were associated with increased risk of early-onset CRC. The same publication also showed that consumption of vegetables, fruits, fish, beta-carotene, and vitamin C had a remarkable protective effect [9].

The aim of our study is to shed more light on potential classic and/or unconventional risk factors that could be contributing to this increased incidence of CRC in the young adult population.

## 2. Materials and Methods

We performed a retrospective large population-based analysis of a commercially available database (Explorys Inc., Cleveland, OH), from the year 1999 to 2019. Explorys is a culmination of Electronic Health Record (EHR) information. Data was collected from 26 reputable healthcare systems of US which were closely integrated [9]. The database constitutes anonymous patient information from those participating institutions which utilize healthcare data gateway (HDG) servers [9]. The servers are protected by firewall within each institution that aggregates deidentified data from different health information systems such as EHR, via billing inquiries. The program Explorys allowed data to be normalized and standardized for further use. Appropriate headings were recorded into the Systematized Nomenclature of Medicine—Clinical Terms (SNOMED-CT), and treatment orders were assigned to SNOMED, which allowed the medication category to be represented. Additionally, RxNorm was utilized to classify the medications themselves. Healthcare organizations that participated in this were assigned an encrypted logon to Explorys which enabled protected data browsing. The data are refreshed at a 24 hour interval with new updates. Explorys is a platform that is compliant with Health Insurance Portability and Accountability Act (HIPAA), and thus further Institutional Review Board (IRB) approval was not required [10].

Patient cohorts were created with Explorys software, and mining of the database was performed on September 1, 2019. The created cohort from this database consisted of every individual who had a diagnosis of primary malignant neoplasm of colon and rectum, between the ages of 20 to 50 years. Furthermore, a control group with patients without the diagnosis of primary malignant neoplasm of colon and rectum within the same age group was established. Demographic data including age, gender, race, and medical comorbidities and social habits including obesity (body mass index (BMI) >30 kg/m^2^), smoking, alcohol intake, diabetes mellitus, hyperlipidemia, Crohn's disease (CD), ulcerative colitis (UC), primary malignant neoplasm of breast, and family history of colorectal cancer were collected.

The overall prevalence of CRC among the study group was calculated by dividing the number of CRC cases by the number of all young adults included in the study. We compared prevalence of CRC in subgroups based on age, gender, and race. The prevalence rates of CRC were calculated for each group by dividing the total number of individuals with CRC by the total number of individuals in the group. The odds ratio, with 95% confidence intervals, was calculated for age, gender, race, obesity (BMI >30 kg/m^2^), tobacco use, alcohol intake, diabetes mellitus, hyperlipidemia, Crohn's disease, ulcerative colitis, primary malignant neoplasm of the breast, and family history of CRC. A multivariate regression model was developed and used to minimize the confounding effects of the previously mentioned covariates. This was attempted with the use of binary logistic regression with colorectal cancer being the outcome. Statistical Package for the Social Sciences (SPSS version 25, IBM Corp.) was the package of choice. In all evaluations, a 2-tailed *P* value of <0.05 was considered statistically significant.

## 3. Results

At the time of data collection, a total of 13,800 patients between the ages of 20 and 50 years had a diagnosis of primary malignant neoplasm of the colon and rectum (Table 1).

**Figure 1:**
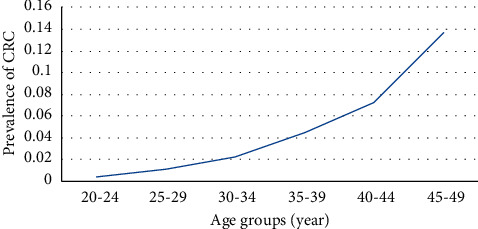
Prevalence of CRC in different age groups.

The prevalence of colorectal cancer increased with age, and the highest rate was seen in the age group of 45–49 (Figure 1).

**Figure 2:**
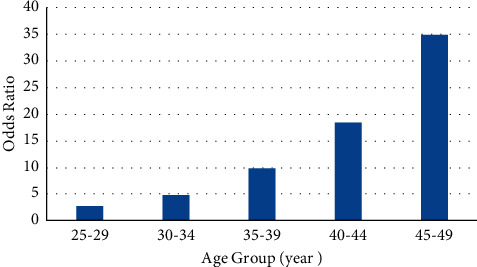
Odds ratio of CRC in different age groups.

The odds ratio of having young-onset colorectal cancer increased with age, reaching 34.89 (95% CI: 29.83–40.82) in the age group of 45–49 years when compared to 20–25 age group as the reference group (Figure 2).

**Figure 3:**
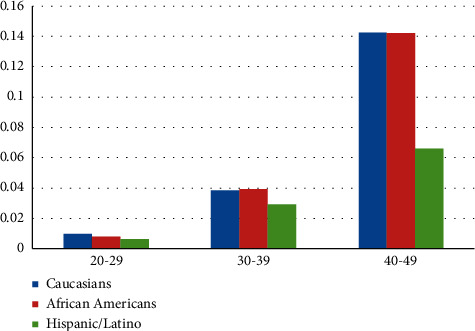
Prevalence of CRC in different race groups.

In terms of ethnicity, CRC was more common among Caucasian and African American young adults, and the prevalence of CRC was lower in the Hispanic and Latino populations compared to other races included in our study, in all age groups (Figure 3).

**Figure 4:**
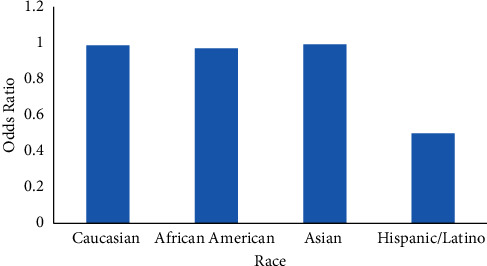
Odds ratio of CRC in different race groups.

Risk of colorectal cancer was lower in the Hispanic/Latino group in comparison with Caucasians (OR: 0.51, CI: 0.44–0.60), and there was no statistically significant difference between Caucasian, African American, and Asian populations (Figure 4).

**Figure 5:**
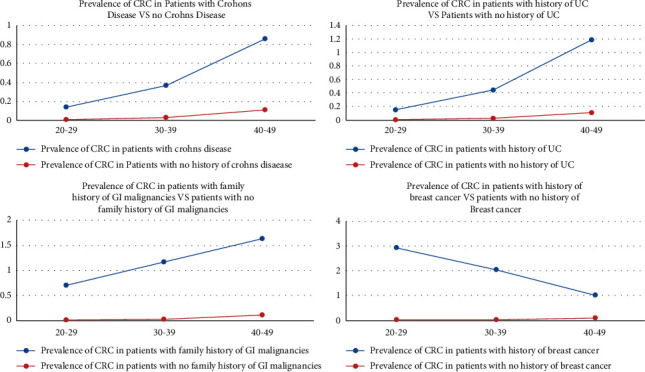
Prevalence of CRC in patients with a history of UC and CD, with and without breast cancer in different age groups.

The prevalence of CRC trended upward with increasing age among patients with family history of GI malignancies and personal history of CD or UC, in comparison to their counterparts in the control group. On the contrary, CRC prevalence declined with age among patients with history of breast cancer, although it slightly increased among patients without breast cancer (Figure 5).

A multivariate analysis of our data revealed that the risk of having young adult-onset colorectal cancer was higher in males with an odds ratio of 1.35 (95% CI: 1.31–1.39).

Family history of colorectal malignancy had the strongest association with young adult colorectal cancer with an odds ratio of 17.78 (95% CI: 16.97–18.62) proceeding with primary malignant neoplasm of breast with odds ratio of 16.94 (95% CI: 15.74–18.24). There was a statistically significant association between UC/CD and young-onset CRC with an odds ratio of 4.42 (95% CI: 4.04–4.84) and 3.7 (95% CI: 3.44–4.07), respectively. In addition, CRC was more common in patients with BMI >30, diabetes mellitus, or hyperlipidemia (OR: 2.48; 95% CI: 2.4–2.57). Among patients with alcohol abuse and nicotine dependence, the odds ratios of developing early-onset colorectal cancer were 1.89 (95% CI: 1.77–2.02) and 1.59 (95% CI: 1.53–1.64), respectively (Table 2).

## 4. Discussion

The definition of “early-onset CRC” or “young adult-onset CRC” remains debatable in literature. The common consensus among the major gastroenterology and oncology societies is that CRC can be considered early onset if the patient's age falls below the screening guidelines [10]. The direct correlation between increasing age and greater incidence of colorectal cancer is well established [1]. We have seen similar trends in our study among patients between ages 20 and 50. With every 5 years of incremental increase in age, the patient's risk of CRC was approximately doubled. We acknowledge that almost half of our cases (48%) with early-onset CRC are between the ages of 45 and 50, which may support American Cancer Society recommendation to start CRC screening for an average-risk people at the age of 45 [4]. However, the topic still remains controversial. As the appropriate targeted strategy of screening and surveillance colonoscopy will decrease the rising incidence and mortality of CRC, [11] American College of Gastroenterology (ACG) recently suggested conditional recommendation to start CRC screening for an average-risk people at the age of 45 [5]. As the GI guidelines remain conditional, we consider that our findings should be extrapolated to <50 age group.

Studies have shown that gender plays a role in the development of colorectal cancer. The CRC incidence and mortality rate are higher in men compared to women. Furthermore, colorectal neoplasia is found at an earlier age in men than in women [12, 13]. In our study, we also found a higher risk of early-onset CRC among male population.

In the United States, the incidence and mortality rates for CRC are higher among African Americans [1]. Reasons for the racial differences are not clear but can be explained by differences in risk factor prevalence in addition to lower rates of access to screening and polypectomy among black population. Moreover, low socioeconomic status among black individuals seems to be a critical factor associated with delayed early screening. A recent epidemiology study showed that the incidence rate of early-onset CRC significantly increased in most racial groups but was stable among African Americans [14, 15]. Because of this trend, the incidence rates of young-onset CRC are equivalent in Caucasian and African Americans now [15]. In our study, we did not find a significant difference in the prevalence of young-onset CRC between Caucasian and African American populations. Furthermore, the prevalence of CRC was lower in Hispanic individuals in comparison to other races in our database. Additionally, the risk of early-onset CRC was almost half for Hispanics in comparison to Caucasian and African American populations. Despite recent studies having shown a rise in CRC among young Hispanic adults, the incidence rate of CRC remains lower in comparison with Caucasian and African American populations [16, 17].

The strength of family history as a risk factor of colorectal malignancy declines as a person ages and is no longer seen after 70 years of age [18]. A European study of risk factors for early-onset CRC showed that family history of colorectal cancer was a greater risk factor (OR: 4.5) in young subjects, versus middle-aged and elderly individuals [9]. We found a stronger direct association between family history of GI malignancy and colorectal cancer among young adults, after excluding FAP and Lynch syndrome. Our database was limited to specify the age of the family members, how many family members have CRC, and if they were first-degree relatives. We also found that the prevalence of CRC increases with age among young adults with the positive family history. The increase was more prominent among individuals between ages 40 and 50. Our findings support the current guidelines to start screening earlier in individuals with family history of CRC [19].

Literature has indicated a potential risk of CRC among patients with history of breast cancer. Two review articles, which both were published in 1994, estimated colorectal cancer relative risks of 1.1 and 1.15, respectively, regarding breast cancer history. These articles concluded that the risk increase was not significant enough to alter colorectal screening protocol in women with history of breast cancer [20, 21]. In addition, multiple studies have suggested higher risk of CRC among patients who developed breast cancer at a younger age. For instance, a systematic review of the literature, including 37 retrospective cohort studies and 8 case-control studies, showed that patients younger than 50 years with breast cancer had an increased risk of developing CRC (OR, 2.5; 95% CI, 1.7–3.5; *P* = 0.001) [22]. It suggested considering CRC screening at the age of 45 in patients with breast cancer <50 years old. In our study, a strong direct association between history of primary malignant neoplasm of breast and early-onset CRC was found. Our finding emphasizes the importance of CRC screening in young adults with history of breast cancer.

Our study showed a higher prevalence of CRC among patients with history of breast cancer in comparison to individuals without such history. Furthermore, prevalence of CRC decreased with age among patients with history of breast cancer in our study population. This trend may indicate that an association exists between breast cancer and development of CRC, which declines with age. Similarly, Newschaffer et al. reported a reduced risk of CRC in women diagnosed with breast cancer after the age of 65 [23]. This trend can be due to a genetic linkage between developing CRC and breast cancer in younger adults. Multiple studies have found controversial results regarding the association between inherited BRCA gene mutations and CRC. Some previous findings have correlated BRCA1 mutation carriers to having a higher risk of colon cancer; however, other studies have failed to replicate and confirm similar results [24–26]. A prospective study of 7015 women younger than 50 years by Sopik et al. reported an almost 5 times increased risk of CRC among BRCA1 mutation carriers [27]. However, they did not find a significant difference between BRCA2 mutation carriers or in elderly females. To account for these findings, a suggestion of initiating CRC screening at the age of 40 with 3 to 5 year intervals until age 50 among BRCA1 mutation carriers was put forth. Then, patients could follow general population guidelines thereafter. In contrary, Mersch et al. did not find an increased risk of CRC among BRCA mutation carriers [28]. MicroRNAs (miRNAs) as regulators of gene expressions have been also studied in different cancers including CRC. Fateh et al. showed that mir-383 was downregulated in CRC samples and suggested it as a potential tumor marker for CRC [29]. More studies with larger sample size will need to be done to find the correlation between miRNAs and CRC pathogenesis.

Our data was consistent with previous studies that showed that patients with inflammatory bowel disease have a greater probability to develop CRC in a younger age, in comparison to sporadic CRC [30–32]. Although the association between UC and colonic neoplasia is well established, there are much fewer studies reporting an increased risk of CRC in individuals with CD. In this study, UC had a stronger association with early-onset CRC than CD. We also found that the prevalence of CRC increases with age in patients with UC and CD. A potential explanation for this finding could be the prolonged inflammatory state in IBD patients.

The association between colorectal cancer and other intestinal diseases such as irritable bowel disease (IBS) and microscopic colitis has been investigated before. In a European study, including 968 IBS patients, only 2 individuals were found to have CRC at the age of 56 and 58 years old [33]. A large case-control study in US showed lower prevalence of colonic adenoma in nonconstipated IBS patients compared with control group [34]. There was no correlation between microscopic colitis and elevated risk of colorectal neoplasia in a US retrospective cohort study [35]. As clinicians are concerned about missing a gastrointestinal pathology, specifically colorectal neoplasia in patients with IBS symptoms, ACG recently recommended colonoscopy in patients with IBS symptoms older than 45 years without warning signs [36].

Multiple studies supported the idea that metabolic syndrome (MS) may be a risk factor for CRC and certain other types of cancers [37, 38]. Each component of MS can increase the risk of CRC through a different pathophysiologic mechanism. In diabetic patients, a compensatory increase in the level of insulin secondary to insulin resistance has been suggested as the possible mechanism of cancer. Hyperinsulinemia leads to increased levels of insulin-like growth factor 1 that acts as an antiapoptotic and growth factor on colonic mucosa, stimulating tumor cell proliferation [39]. Obesity, as a component of MS, is associated with chronic inflammatory state due to the production of proinflammatory cytokines, such as TNF-*α* and IL-6. Chronic inflammation is the possible mechanism of CRC in obese patients [40]. Data regarding the association between dyslipidemia and CRC have been controversial. Yao et al., in a meta-analysis of 17 prospective studies, showed an increased risk of CRC among patients with high triglyceride and high total cholesterol [41]. In contrast, the Framingham study showed an inverse correlation between colon cancer and serum cholesterol levels [42]. The carcinogenic effect of bile salts on colonic epithelial cells is hypothesized as the mechanism of CRC, in patients with high fat intake [43]. Among the components of metabolic syndrome, hyperglycemia and obesity have been considered the strongest risk factors for CRC [44–47]. In this study, a higher risk of early-onset CRC among patients with history of diabetes mellitus, hyperlipidemia, or BMI >30 kg/m^2^ has been established. Our data in conjunction with previous studies indicate that the rise of young-onset CRC can be partially attributed to the increased incidence and prevalence of MS, in particular within the developed countries and this age group [48].

Direct dose-related correlation between smoking, alcohol use, and CRC incidence has been shown in previous studies [49–51]. Although we could not determine the duration, amount of smoking, and alcohol use in our population, the risk of CRC was greater in tobacco and alcohol dependent patients. Microsatellite instability in smokers and DNA hypomethylation in alcoholics have been suggested as possible mechanisms of colonic carcinogenesis [52, 53].

The association between diet and CRC has been investigated before. A European case-control study showed that the diet centered on high levels of processed meat was associated with increased risk of early-onset CRC [9]. The same publication also showed that consumption of vegetables, fruits, fish, beta-carotene, and vitamin C had a remarkable protective effect. Similarly, consumption of *Allium* vegetables such as garlic reduces the crypt foci and adenomatous polyp as the precursor lesions for colon cancer [54]. The *Allium* extract also showed promising therapeutic results in patients with CRC.

Although Explorys provides information from 26 major health systems across the nation, it has several limitations that should be noted in our study. This cohort study shows the validity and comprehensiveness of the results to be directly related to the EMR in use at each institution. Furthermore, in converting ICD-9 or CPT codes to equivalent Explorys descriptors, possibilities for redundancy and ambiguity are introduced. Additionally, data regarding family history was limited. It did not specifically determine if it was first- or second-degree relation and how many family members had been affected. Explorys also broadly reports family history of GI malignancy not specifically CRC. In addition, we also did not have the data of BRCA mutation carriers in the database. Lastly, other potential risk factors such as medications, activity level, and diet could not be reliably tracked in this database.

## 5. Conclusion

This study is one of the most current yet comprehensive papers in the analysis of risk factors for early-onset CRC. As expected, previously known risk factors such as family history of CRC and individual history of IBD showed a strong correlation with early-onset CRC. Additionally, the individual history of breast cancer in our study has shown a noteworthy association between breast cancer and CRC, which has the potential to warrant an early screening protocol in this subset as well. To a lesser extent, other risks such as obesity, diabetes mellitus, hyperlipidemia, alcohol abuse, and smoking have shown an association with early-onset CRC. This study once again emphasizes the importance and merits of early screening and identification following identification of high-risk individuals. Our findings also emphasize the importance of lifestyle modification that may help overcome multiple risk factors in the prevention of CRC. Follow-up studies may provide further clarification on this aspect.

## Figures and Tables

**Table 1:** Patients' characteristics.

	Patients with CRC	Patients with no history of CRC
	*N* = 13800	*N* = 26906160
	0.051%	99.9%
20–24 (age)	150 (1%)	3764170 (14%)
25–29 (age)	480 (3%)	4470380 (17%)
30–34 (age)	990 (7%)	4835050 (18%)
35–39 (age)	2030 (15%)	4864490 (18%)
40–44 (age)	3500 (25%)	4479380 (17%)
45–49 (age)	6650 (48%)	4492690 (17%)
Female (gender)	7015 (51%)	15004190 (56%)
Male (gender)	6785 (49%)	11901970 (44%)
African American (race)	1990 (14%)	3203660 (12%)
Asian (race)	290 (2%)	478440 (2%)
Asian/Pacific Islander (race)	60 (0%)	88740 (0%)
Caucasian (race)	9390 (68%)	14266020 (53%)
Hispanic/Latino (race)	170 (1%)	500240 (2%)
Tobacco user	2990 (22%)	3011180 (11%)
Hyperlipidemia	2500 (18%)	1367580 (5%)
Diabetes mellitus	1760 (13%)	857990 (3%)
Family history of CRC	1380 (10%)	95980 (0%)
Body mass index >30	1090 (8%)	503840 (2%)
Alcohol abuse	470 (3%)	440980 (2%)
Primary malignant neoplasm of breast	340 (2%)	35300 (0%)
Inflammatory bowel disease	570(4%)	122870 (0%)

**Table 2:** Predictors of CRC in young adults.

	OR	95% CI
Males vs. females	1.355	1.317 to 1.394
Alcohol abuse	1.895	1.778 to 2.021
Smoking	1.592	1.539 to 1.647
Body mass index >30, diabetes mellitus, or hyperlipidemia	2.485	2.400 to 2.572
Primary malignant neoplasm of breast	16.947	15.740 to 18.248
Ulcerative colitis	4.427	4.049 to 4.840
Crohn's disease	3.744	3.441 to 4.073
Family history of colorectal cancer	17.781	16.974 to 18.627

All *P* values < 0.0001.

## Data Availability

The data supporting the findings of this study are included within the article and at the IBM Explorys Platform.
